# Life Satisfaction and Online-Gambling Communities: A Cross-National Study of Gambling Activities Among Young Finnish, American, South Korean and Spanish People

**DOI:** 10.1007/s10899-021-10081-8

**Published:** 2021-10-18

**Authors:** Aki Koivula, Atte Oksanen, Anu Sirola, Iina Savolainen, Markus Kaakinen, Izabela Zych, Hye-Jin Paek

**Affiliations:** 1 Department of Social Research, University of Turku, Assistentinkatu 7, 20014 Turku, Finland; 2 Faculty of Social Sciences, Tampere University, Tampere, Finland; 3 Institute of Criminology and Legal Policy, University of Helsinki, Helsinki, Finland; 4 Department of Psychology, University of Córdoba, Córdoba, Spain; 5 Department of Advertising & Public Relations, Hanyang University, Ansan, Republic of Korea

**Keywords:** Online communities, Problem gambling, Online-gambling communities, Online networks, Life satisfaction

## Abstract

Gambling is a potential hazard to life satisfaction, yet peer relationships online might buffer this risk. This study analyzed the ways problem gambling is associated with life satisfaction as well as the extent to which the use of online-gambling community participation and, alternatively, offline belonging affect this association. A web-based survey was conducted among people aged 15–25 in Finland (*n* = 1,200), the United States (*n* = 1,212), South Korea (*n* = 1,192), and Spain (*n* = 1,212). The main variables included life satisfaction, problem gambling measured by the South Oaks Gambling Screen, online-gambling community participation, and offline belonging. Controls included compulsive internet use, hazardous drinking, psychological distress, income, age, and gender. Linear regression models were employed with country interactions. Results showed problem gambling had a negative relationship with life satisfaction, but the association was explained by control variables. Online-gambling community participation had a positive relationship with life satisfaction, especially among pathological gamblers who had poor offline relationships. Country comparisons revealed that the direct effect of excessive gambling and the compensating effect of online-gambling communities were most prominent in Finland.

## Introduction

Gambling has always provided people with excitement, enjoyment, and fun as a pastime activity (Bloch, 1951), but it also has important gross market value across countries (Orford, 2019). However, problem gambling is the downside of this pleasure-producing activity, generating undesirable costs that potentially lead to the reduction of human well-being (Farrell, 2018; Tang & Oei, 2011; Wanner et al., 2006). When it becomes a problem, gambling begins to have a major influence on an individual’s life, his or her close ones, and eventually, society as a whole (Blinn-Pike et al., 2010; Gainsbury et al., 2014). Problem gambling leads to negative outcomes, such as loss of money and consequent financial difficulties, and damages close relationships (Oksanen et al., 2018; Orford, 2010). Problem gambling is considered a behavioral addiction that typically begins to develop in early adolescence (Derevensky et al., 2019; Orford, 2010).

The digitalization of gambling has fundamentally changed the manifestation of and opportunities for gambling (Gainsbury, 2015; Gainsbury et al., 2013). Gambling, characterized by wagering and betting mechanisms involving real money, has significantly increased due to online gambling sites, such as online poker rooms (McBride & Derevensky, 2009). Without the schedule and location-based restrictions of offline gambling, online gambling is accessible to a broader group of potential players (Torres & Goggin, 2014), even underage individuals, with 24/7 access from mobile phones and other portable devices. Online gambling utilizes increasing amounts of online gaming characteristics, including graphics, animation, and playability, to make the gambling activity more attractive (Albarrán-Torres & Apperley, 2018; Sirola et al., 2020). These characteristics, along with other unique features of gambling (e.g., entertainment value and a perceivably easy way to make money), have increased the popularity and attractiveness of online gambling. Younger individuals, for whom using technology and digital services is already a major part of daily life, are participating in online gambling at an increasing rate (Elton-Marshall et al., 2016; Parke & Griffiths, 2011).

Due to its addictive nature and the risk of losing large amounts of money, gambling may become pathological and cause severe problems related to young individuals’ lives and well-being. Studies have shown a strong relationship exists between gambling and unhealthy behaviors (e.g., Griffiths & Barnes, 2008; Kairouz et al., 2012; Wood & Williams, 2011). However, previous research has provided little insight into the way gambling affects the subjective well-being of young people. In general, life satisfaction, an individual’s subjective assessment of his or her entire life, is a central part of subjective well-being (Busseri & Sadava, 2011). It continues to be an essential part of social science research concerned with evaluating desirable circumstances for well-being through individual satisfaction (Kouvo & Räsänen, 2015; Proctor et al., 2009; Schiffrin & Nelson, 2010). We assume that excessive gambling is associated with lower life satisfaction by compounding multiple factors associated with negative emotions and evaluations of one’s life situation.

Previous research has indicated that using diverse digital resources, including social media, is positively associated with life satisfaction (Huang et al., 2021; Keipi et al., 2018; Lissitsa & Chachashvili-Bolotin, 2016; Valenzuela et al., 2009). In addition, belonging to social groups is highly important for well-being (Ballas & Dorling, 2007; Baumeister & Leary, 1995). Furthermore, people are more likely to form and value social ties with others who share their attitudes and interests (McPherson et al., 2001), and this tendency is especially salient in social media (Hartl et al., 2015; Kaakinen et al., 2020). In line with these findings, research has reported that young problem gamblers tend to form new gambling-related relationships at the expense of previous friendship ties (Blinn-Pike et al., 2010). Drawing on this finding, we consider that participation in online gambling-related communities could be a modifying factor enmeshed in young people’s gambling activities and life satisfaction. Online network sites, such as Facebook and Instagram, capture the social and expressional components of human interaction, and they have become central to social interaction across the Western world. Notably, platforms are structured uniquely to enable specific uses. Previous research has highlighted that online-gambling communities are attractive to individuals who are interested in gambling overall and who experience psychosocial problems (Sirola et al., 2018, 2019).

The literature currently lacks studies that analyze online gambling-related behavior and life-satisfaction among young people globally. Although risk behaviors such as gambling may have negative consequences for subjective well-being, we do not know the mechanisms of other online gambling-related activities. Even though gambling may carry risks, the social aspects of gambling, such as participating in online-gambling communities, might also have positive meanings for young people. Analogically, it could be also expected that online-gambling communities could have a positive role in seeking social ties. Our study objective is to analyze the relationship between online gambling and life satisfaction by comparing young individuals from four countries: Finland, the United States, South Korea, and Spain. Additionally, we examined whether participation in gambling communities compensates for subjective dissatisfaction resulting from gambling problems.

## Problem Gambling and Life-Satisfaction

The concept of *life satisfaction* refers to an individual’s subjective feelings about his or her life (Diener, 1984). Life satisfaction can be distinguished from the concept of happiness because it involves a more holistic view of life, rather than focusing solely on individual moments that produce good feelings and emotions (Diener et al., 1985). In this respect, life satisfaction is an appropriate measure to evaluate the consequences of problem gambling linked to a variety of factors that impair life satisfaction. First, problem gambling is likely to weaken financial and material resources, which are part of an individual’s subjective well-being (Callan et al., 2015; Nickerson et al., 2007; Tabri et al., 2015). Problem gambling also has psychological propensities similar to addiction. These include the tendency to experience dissatisfaction with one’s life situation (Headey et al., 1993; Hodgins et al., 2007). In addition, problem gambling is typically associated with behavioral disorders such as substance abuse and compulsive internet use (Petrty, 2001; Van Rooij et al., 2017), which may eventually be reflected in decreased subjective well-being as well.

Drawing on previous studies, problem gambling and life satisfaction depend on similar demographic factors, but the direction of the effects can be different. For example, problem gambling has been found to increase significantly at age 18 (Welte et al., 2011), but the development of life satisfaction has been observed to follow a U-shaped curve; that is, satisfaction decreases toward middle age and then increases later in life (Blanchflower & Oswald, 2008). Studies have found that young women have a greater tendency to be dissatisfied with their lives than men (Moksnes & Espnes, 2013), but young men are more likely than women to become problem gamblers (McCormack et al., 2014). However, in addition to the significant financial risks involved, problem gambling is generally more likely to occur among those belonging to lower income groups (Canale et al., 2017), who are also, on average, less satisfied with their lives (Nickerson et al., 2007).

Social interactions and close relationships are central to the well-being of an individual (Ballas & Dorling, 2007). Previous research has shown that social activity and well-functioning relationships predict higher levels of life satisfaction (Lim & Putnam, 2010). In this respect, problem gambling may decrease satisfaction because it has been found to isolate individuals from their close relationships (Shaw et al., 2007). For example, previous studies have found that problem gambling damages social relationships with family and friends (Downling et al., 2014). Similar to other behavioral addictions, gambling reduces the amount of time one can spend on maintaining social relationships (Wood & Griffiths, 2007). Problem gambling is also a stigmatized behavior. Accordingly, shame caused by one’s problem gambling can lead to social isolation (Horch & Hodgins, 2008).

One possible factor for dissatisfaction is the loneliness that often results from social isolation. Indeed, previous studies have shown that single people, for example, have a greater risk of being problem gamblers (Botteril et al., 2016). On the other hand, gambling can also substitute social acceptance, which has a positive effect on the well-being of individuals. To this effect, previous studies have shown that working-class women tend to gamble because they can meet other women of the same socioeconomic status while gambling (Dixey, 1996; Trevorrow & Moore, 1998).

## Online-Gambling Communities and Life Satisfaction

Online communities develop through computer-mediated communication, such as discussion forums or chatrooms, and typically form around shared interests and identities (Komorowski et al., 2018; McEwen & Wellman, 2013; Mikal et al., 2016; Parke & Griffiths, 2011; Wellman & Gulia, 1999). Given their various social features, online communities have an increasing potential to provide individuals with support and well-being (Liu et al., 2020). Examples of shared interests around which online communities are formed include diet and nutrition, self-help, hobbies, and professional skills (Huang et al., 2019; Tanis, 2007). Previous studies have shown that various gaming communities can be important for an individual’s social capital (Molyneusx et al., 2015). In this respect, it is essential to note that the quality of online interaction matters in establishing social support and psychological well-being rather than mere frequency or amount of social media use (Oh et al., 2014).

Studies have also shown online-gambling communities are generally international platforms that include gaming and gambling discussions (Sirola et al., 2020). Sirola et al. (2018) found that young Finnish problem gamblers preferred using communities that include discussions on ways to facilitate and improve gambling skills and experiences, and only a few reported utilizing problem-gambling-oriented communities with the aim of seeking help and recovery. However, other studies have shown the existence of a variety of online self-help groups such as discussion forums for problem gamblers (Mudry & Strong, 2013; Rodda et al., 2018; Wood & Wood, 2009). Communities of people who seek problem-gambling recovery are found helpful in dealing with gambling-related problems and in feeling less alone with one’s problems (Wood & Wood, 2009). Online-gambling communities are also more popular among lonely people who gamble excessively (Sirola et al., 2019). Thus, gambling-related online communities can compensate for the social exclusion associated with problem gambling and, consequently, improve an individual’s life satisfaction. Although online communities can encourage individuals to gamble more often and help maintain and normalize problematic gambling habits, they may also offer important social ties, support, and sense of belonging to their participants, potentially increasing subjective well-being and life satisfaction.

## Hypothesis Development

Our first hypothesis was that young problem gamblers are less satisfied with life than other young people are (Hypothesis 1). Many factors involved in problem gambling predict low levels of life satisfaction, such as isolation from offline social relations (Wang & Wang, 2013), economic problems (Oksanen et al., 2018), and poor mental health (Petry & Weinstock, 2007). By drawing on existing literature, we assumed the relationship between problem gambling and life satisfaction is related to demographic factors (e.g., age, gender, and income) and behavioral factors (e.g., psychological distress and social belonging), with significant confounding effects on online gambling and life satisfaction.

Next, we focused on the effects of gambling-related online communities on life satisfaction. The starting point of the study was that participation in online communities could increase life satisfaction (Keipi et al., 2018; Valenzuela et al., 2009). This has been particularly evident in social networking sites focused on hobbies (Mahan et al., 2015) or those that provide social support (Oh et al., 2014; Liu et al., 2020). Accordingly, we assumed that online-gambling community participation has a direct positive relationship with life satisfaction (Hypothesis 2). Additionally, we assumed that the association of online-gambling community participation is particularly strong for probable pathological gamblers (Hypothesis 3).

By drawing on the recent literature on social media networks and communities, we considered that online communities could be helpful and supportive for those experiencing poor social relationships (Matook et al., 2015; Pittman, 2015). Accordingly, we assumed that online-gambling community participation was associated with life satisfaction among problem gamblers who have poor offline relationships in terms of belonging (Hypothesis 4). The rationale here is that if a person has good relations with his or her close ones, then the influence of online-gambling communities is likely less significant in terms of life satisfaction. Instead, online communities can offset the benefits of social relationships to life satisfaction despite poor offline relationships.

In addition to the established hypotheses, we examined differences between countries. In general, problem gambling is associated with similar factors in these countries. Men, low-income earners, substance abusers, and people with mental problems are the most affected by gambling problems (Castren et al., 2013; Chóliz et al., 2021; Welte et al., 2017; Williams et al., 2013). We further assume that the different underlying factors related to gambling activities may exist at the country level. According to Sirola et al. (2019), weak offline relationships are likely to increase the tendency of Finnish players to seek out online communities, which in turn increase their gambling activities. In this respect, online communities can harm life satisfaction. On the other hand, it has been found that in the United States, these communities can also provide players social support, which may be reflected positively in life satisfaction (Sirola et al., 2020). Therefore, we could not directly specify the direction of the hypothesized differences between countries.

## Method

### Participants

The participants were 15–25-year-old adolescents and young adults from Finland (*n* = 1,200; 50% female; *M*_age_ = 21.29; *SD*_age_ = 2.85; entered the study in March–April 2017), the United States (*n* = 1,212; 50.17% female; *M*_age_ = 20.05; *SD*_age_ = 3.19; entered January 2018), South Korea (*n* = 1,192; 50.42% female; *M*_age_ = 20.61; *SD*_age_ = 3.24; entered February 2018), and Spain (*n* = 1,212; 48.76% female; *M*_age_ = 20.07; *SD*_age_ = 3.16; entered January 2019). To achieve data that mirror the current population estimates of each examined country, we pre-stratified each sample according to age, gender, and location.

### Data Collection

Data collection was performed by employing comprehensive web-based surveys. The participants were recruited from a volunteer participant pool administered by Dynata (formerly known as Survey Sampling International). The study was designed in Finnish and English. The survey mostly consisted of previously validated measures that have been widely used in comparative research, and the back-translation process guaranteed the accuracy and comparability of other items. In 2018, the data collection expanded to South Korea and Spain, where proficient native Korean and Spanish speakers translated the English survey into Korean and Spanish. These surveys went through the back-translation procedures to assure the study was internally consistent, and the items matched accurately.

### Measures

We used life satisfaction as the dependent variable. The survey used a single-item to measure life satisfaction by asking, “All things considered, how satisfied are you with your life?” Respondents rated their subjective feelings on a scale from 1 (*extremely dissatisfied*) to 10 (*extremely satisfied*). Even though life satisfaction can be measured with multiple statements by utilizing, for example, the Satisfaction With Life Scale (Diener et al., 1985), the single-item measure produces similar results to those achieved with longer scales (Cheung & Lucas, 2014).

As for independent variables, we used problem gambling, gambling-community participation, and offline belonging. Problem gambling behavior was measured by using the South Oaks Gambling Screen scale (SOGS). The SOGS is based on *Diagnostic and Statistical Manual of Mental Disorders* (3rd ed.; *DSM-3*) and *DSM-4* criteria for pathological gambling and it is among the most widely used screening tools for problem gambling (Lesieur & Blume, 1987). In addition to *DSM-3* and *DSM-4* criteria, the measure was also reported to have a strong correlation with *DSM-5* symptoms (Goodie et al., 2013). We followed Castrén et al. (2013) and Oksanen et al. (2018) and used the following cutoffs to categorize the variable: 0–2 = *no problem gambling*, 3–4 = *at-risk gambling*, ≥ 5 = *probable pathological gambling*. In the interaction analysis, we used a binary variable that differentiated probable pathological gamblers from the rest of the participants.

Gambling-community participation was measured with a question similar to that used in Sirola et al. (2019) : “How often do you use gambling-related discussion forums or communities?” The responses were given on a scale including *never*, *seldom*, *daily*, and *many times a day*. To guarantee enough observations from each category, we combined options of *daily* and *many times a day* to have a 3-point categorical variable in the analysis.

We analyzed the effects of offline belonging to priority groups using similar measurement as many previous studies that have addressed young people’s online behavior (e.g., Miller et al., 2021; Savolainen et al., 2020) . We used a composite variable that was combined from three items: “How strongly do you feel you belong to each of the following: (a) family, (b) friendship group, (c) school or work community?” The scale ranged from 1 (*not at all*) to 10 (*very strongly*). Generally, we utilized the variable as a continuous variable, but we used a categorized variable to find more interpretable results in interaction analysis. In the categorization, we first rounded the decimals to the nearest whole number, after which we classified the respondents who received a value of 1–3 into the low category, those who received 4–7 into the medium category, and those who received 8–10 into the high category.

Control variables were included by following the previous studies on life satisfaction and gambling behavior. We controlled for age, gender, financial situation, psychological distress, compulsive internet use, and hazardous alcohol use. Age was used as a continuous variable in the models. We also included a self-reported measure of income by considering respondents’ average money they had available every month after the necessary expenses (1 = *less than 50 dollars*; 2 = *50–100 dollars*; 3 = *101–200 dollars*; 4 = *201–300 dollars*; 5 = *301–500 dollars*; 6 = *501–1000 dollars*; 7 = *more than 1,000 dollars*). Psychological distress was measured with the widely used 12-item General Health Questionnaire. The measure evaluates respondents’ current state of psychological well-being with questions such as, “Have you recently felt constantly under strain” (Goldberg et al., 1997; Pevalin, 2000). To control other addictive behaviors, we considered compulsive internet use and hazardous drinking. We used the 14-item Compulsive Internet Use Scale to measure compulsive internet use (Meerkerk et al., 2009). Finally, we used the Alcohol Use Disorders Identification Test to measure hazardous drinking.

The descriptive statistics of applied variables are presented in Table 1.

**Table 1:** Descriptive statistic of the applied measures

Categorical variables:	*n*	%
*Problem gambling (SOGS)*		
No problem	3,966	82.4
At risk	415	8.6
Probable pathological	435	9.0
*Online-gambling community participation*		
Never	4,079	84.7
Seldom visitor	445	9.2
Daily visitor	292	6.1
*Gender*		
Male	2,416	50.2
Female	2,400	49.8
*Country*		
Finland	1,200	24.9
US	1,212	25.2
South Korea	1,192	24.8
Spain	1,212	25.2

Continuous variables:	Mean	SD	Range	
Offline Belonging	6.8	2.1	1	10
Age	20.5	3.2	15	25
Income level	3.2	1.8	1	7
Hazardous drinking (Alcohol Use Disorders Identification Test)	2.7	2.7	0	12
Compulsive internet use (Compulsive Internet Use Scale)	21.5	12.7	0	56
Psychological distress (12-item General Health Questionnaire)	13.8	6.4	0	36

Observations: 4,816

### Analytical Strategy

We began the analysis by estimating the direct association between problem gambling and life satisfaction while the country-level differences were fixed by adding country as a factor variable in the model. In addition, the control variables were introduced stepwise into the model. Afterwards, we analyzed whether these associations exist in different countries and added the interaction term *country* × *problem gambling* to run the model with all other variables.

The second part of the analysis began with an analysis of the direct association between gambling-community participation and life satisfaction, whereas the country-level effects and control variables were considered. Then, we assessed the ways community participation moderated the effect of problem gambling on life satisfaction by adding the interaction term *problem gambling* × *gambling-community participation* into the model.

Finally, we considered the last hypothesis (Hypothesis 4) and examined whether the effect of gambling-community participation is relatively strong among those problem gamblers who had poor offline relationships. More specifically, we analyzed the moderating effect of offline relationships and introduced a three-way interaction term between the factors of *gambling-community participation* × *problem gambling* × *offline belonging.* We also described these associations in different countries.

The paper utilized ordinary least squares (OLS) regression throughout the analysis to account for the prediction of life satisfaction according to various continuous and categorical variables. We also performed different interaction analyses using the same method while control variables were held as constant as possible. We report standardized coefficients (*β*) and unstandardized regression coefficients (*B*) along with their standard error and statistical significance (*p* value) estimates. In addition, the interaction effects were elaborated into more interpretable forms with graphs. The regression analyses were performed with STATA 16 and the user-written coefplot package was utilized to illustrate the results into the graphs (Jann, 2014).

## Results

Table 2 presents the results of the regression analysis in which we analyzed the ways problem gambling is associated with life satisfaction. Problem gambling had a negative relationship with life satisfaction (*B* = − 0.31, *p* < 0.01). The second model indicated the demographic or economic factors did not confound the relationship between problem gambling and life satisfaction, even though age and income were significant predictors. Models 3–5 suggested that the link between problem gambling and life satisfaction was associated with compulsive internet use, psychological distress, and offline belonging. After controlling for these behavioral variables, the effect of problem gambling was not significant. The beta values estimated from the final model indicated offline belonging was the most contributive variable in the final model.

**Table 2:** Predicting life satisfaction by problem gambling severity and confounding variables

	M1	M2	M3	M4	M5	M5
Variables	B (SE)	B (SE)	B (SE)	B (SE)	B (SE)	β
*Independent variable: Gambling problem*						
No problem (omitted)						
At− risk	− 0.22*	− 0.28*	− 0.20	− 0.01	0.07	0.01
	(0.11)	(0.11)	(0.11)	(0.10)	(0.09)	
Probable pathological	− 0.31**	− 0.40***	− 0.27*	− 0.01	0.12	0.02
	(0.11)	(0.11)	(0.11)	(0.10)	(0.09)	
*Control variables:*						
Age		− 0.10***	− 0.10***	− 0.06***	− 0.02*	− 0.03
		(0.01)	(0.01)	(0.01)	(0.01)	
Female		− 0.20**	− 0.19**	0.10	0.08	0.02
		(0.06)	(0.06)	(0.06)	(0.05)	
Income		0.12***	0.13***	0.09***	0.07***	0.06
		(0.02)	(0.02)	(0.02)	(0.01)	
Hazardous drinking			− 0.01	0.03**	− 0.00	− 0.00
			(0.01)	(0.01)	(0.01)	
Compulsive internet use			− 0.01***	0.01***	0.01*	0.03
			(0.00)	(0.00)	(0.00)	
*Psychological distress*				− 0.16***	− 0.09***	− 0.28
				(0.00)	(0.00)	
*Offline Belonging*					0.50*** (0.01)	0.47
*Country effects:Finland (omitted*						
US	0.04	− 0.07	− 0.05	− 0.11	− 0.07	− 0.01
	(0.09)	(0.09)	(0.09)	(0.08)	(0.07)	
South Korea	− 0.83***	− 0.90***	− 0.84***	− 0.96***	− 0.85***	− 0.17
	(0.09)	(0.09)	(0.09)	(0.08)	(0.07)	
Spain	0.02	− 0.08	− 0.06	− 0.10	− 0.24***	− 0.05
	(0.09)	(0.09)	(0.09)	(0.08)	(0.07)	
Constant	6.42***	8.42***	8.61***	9.19***	4.35***	4.35***
	(0.06)	(0.24)	(0.24)	(0.22)	(0.23)	(.)
Observations	4,816	4,816	4,816	4,816	4,816	4,816
R-squared	0.03	0.05	0.05	0.24	0.42	0.42

Standard errors in parentheses

^*^ p < 0.05

^**^ p < 0.01

^***^ p < 0.001

**Fig. 1:**
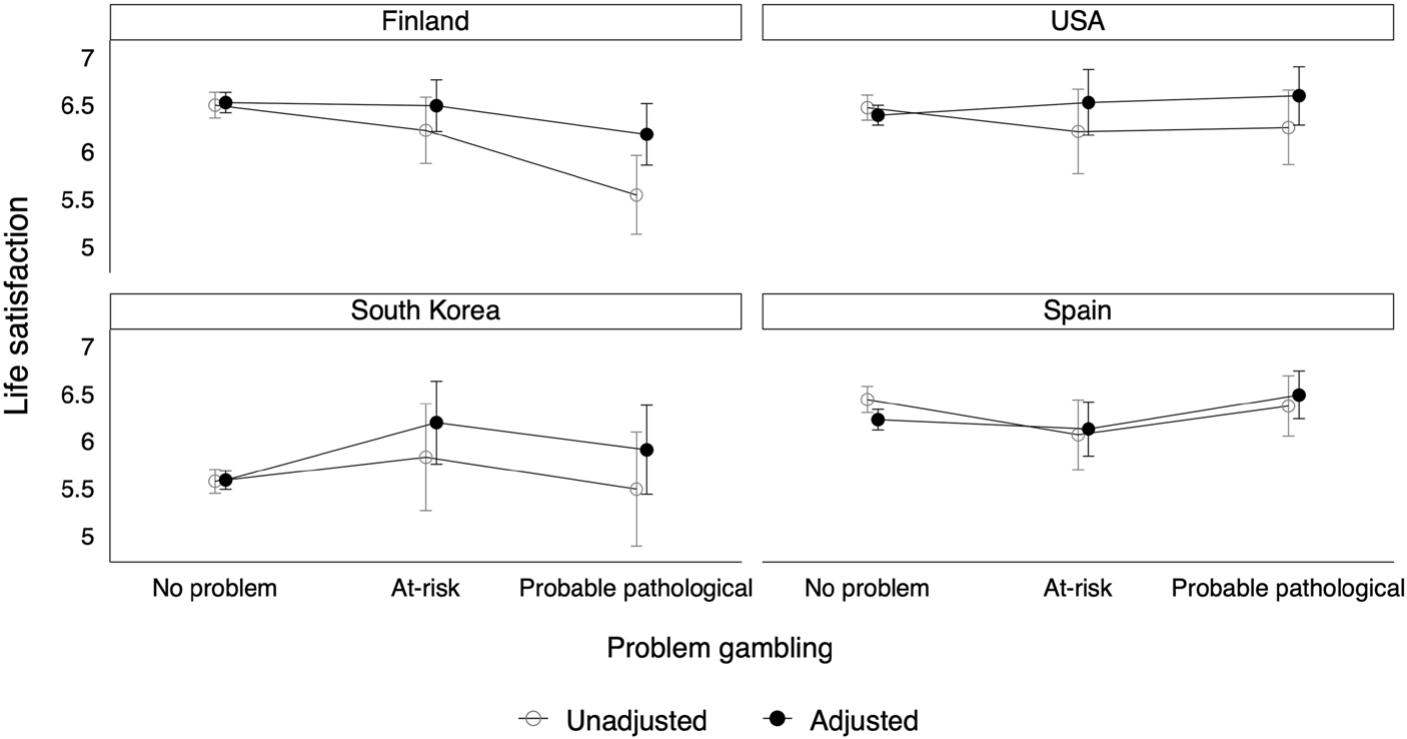
Life satisfaction according to problem gambling severity by country, unadjusted and adjusted predictive margins with 95% confidence intervals from the OLS models presented in Table 4

Figure 1 shows country-level interactions that were estimated from regression analysis. It was found that Finland was the only country where problem gambling had a negative relationship with life satisfaction. In South Korea, young people were generally less satisfied, and online gambling did not affect this result. Problem gamblers in Finland and Korea were almost equally happy/dissatisfied with their lives, whereas Finns were better off when the non-problem gamblers were compared across countries.

The second section analyzes the ways online-gambling community participation is associated with life satisfaction and how they explain the life satisfaction of probable pathological gamblers. We continued the analysis by adding the gambling-community participation into the final model presented in Table 2. The results revealed that people who visited gambling communities every day were more satisfied with their lives (*B* = 0.52, *p* < 0.001). The effects of control variables were similar to those presented in the previous models in Table 2. The results also revealed the interaction between gambling-community participation and problem gambling (*B* = 0.64,* p* < 0.001), which indicates that those gamblers who used gambling communities were more satisfied with their lives.

**Fig. 2:**
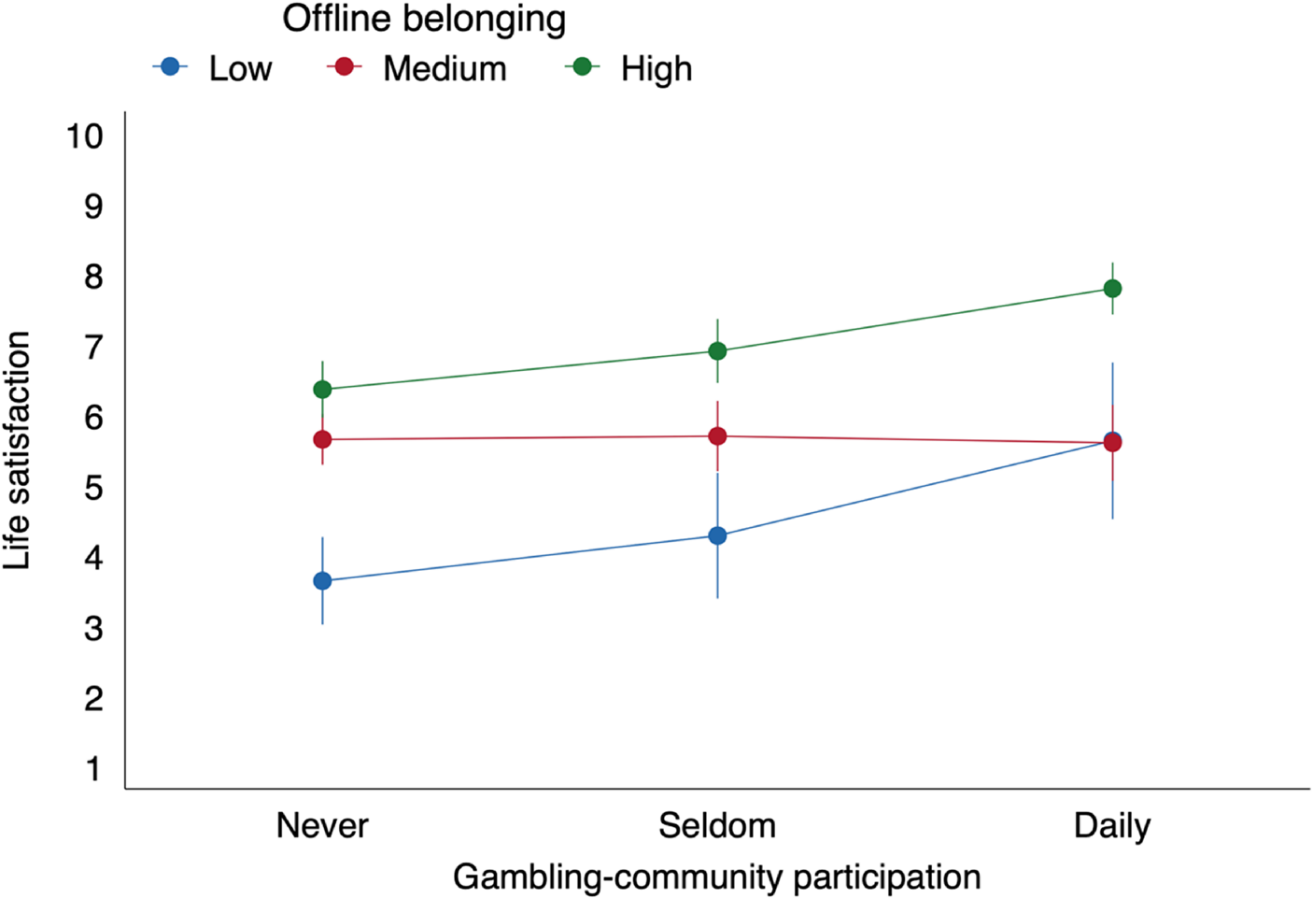
Life satisfaction according to use of gambling communities and offline belonging among probable pathological gamblers, adjusted predictive margins with 95% confidence intervals from the OLS models presented in Table 3

Finally, we checked the extent to which offline relationships moderated the effects of gambling-related activities. The final model confirmed our fourth hypothesis. We found that the effect of online-gambling community participation was less prominent among those gamblers who reported high offline belonging (*B* =  − 2.60,* p* < 0.001). The interaction also explained a significant proportion of the variance in life satisfaction produced by the use of online communities. Figure 2 indicates that the effect of online communities is relatively higher among those who reported low belonging to offline relationships when compared to those with medium or high belonging to offline relationships. What is important to notice in the figure is that the difference between offline belonging groups (low, medium, and high) diminished when we considered the effect of gambling-community participation (Table 3).

**Table 3:** Predicting life satisfaction according to participation in online gambling- communities, probable pathological gambling and offline belonging, unstandardized regression coefficients with standard errors

	M1		M2		M3	
	B	SE	B	SE	B	SE
*Independent variables*						
*Online-gambling community participation*						
Never (omitted)						
Seldom visitor	0.14	(0.09)	0.03	(0.02)	0.02	(0.00)
Daily visitor	0.52***	(0.11)	0.30	(0.30)	− 1.47***	(0.44)
*Problem gambling*						
No problem / At risk (omitted)						
Probable pathological	− 0.07	(0.10)	− 0.29*	(0.13)	− 0.73*	(0.33)
*Offline belonging*						
Low belonging (omitted)						
Medium belonging	1.32***	(0.08)	1.32***	(0.09)	1.25***	(0.10)
High belonging	2.61***	(0.08)	2.59***	(0.09)	2.51***	(0.10)
*Interaction effects*						
Seldom visitor						
X Probable pathological			0.29	(0.23)	0.54	(0.63)
Daily visitor						
X Probable pathological			0.65**	(0.24)	3.47***	(0.79)
Seldom visitor						
X High belonging						
X Probable pathological					− 0.05	(0.71)
Daily visitor						
X High belonging						
X Probable pathological					− 2.60**	(0.81)
*Country effects*						
Finland (omitted)						
US	− 0.05	(0.07)	− 0.05	(0.07)	− 0.06	(0.07)
South Korea	− 0.87***	(0.07)	− 0.88***	(0.07)	− 0.88***	(0.07)
Spain	− 0.22***	(0.07)	− 0.22***	(0.07)	− 0.22***	(0.07)
Constant	6.12***	(0.22)	6.16***	(0.22)	6.31***	(0.22)
Observations	4,816		4,816		4,816	
R-squared	0.38		0.38		0.38	

Models control for Age, Gender, Income, Hazardous drinking, Compulsive internet use, Psychological distress

Standard errors in parentheses

^*^ p < 0.05

^**^ p < 0.01

^***^ p < 0.001

**Fig. 3:**
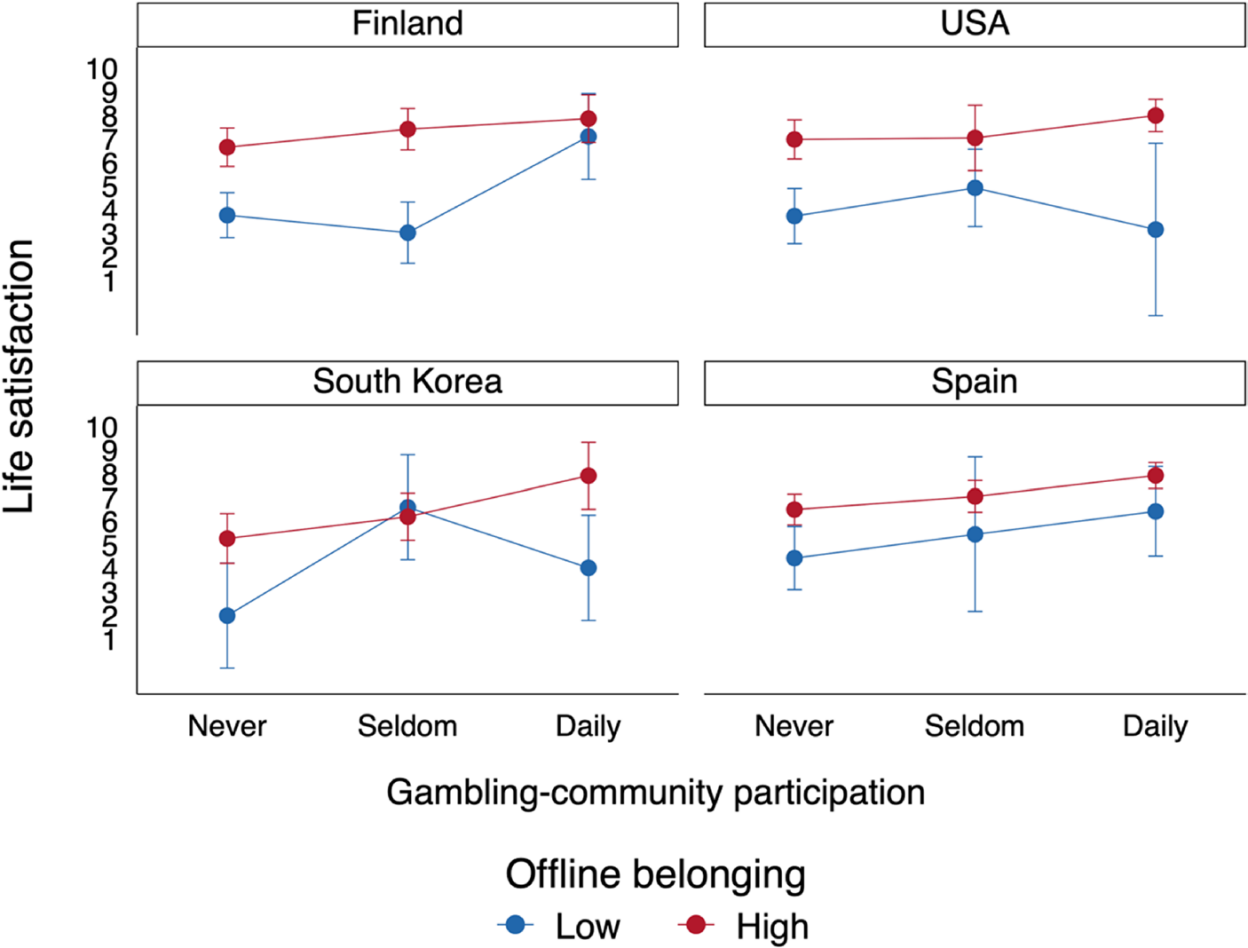
The effect of gambling-community participation among the probable pathological gamblers according to the level of offline belonging by country. Adjusted predictive margins with 95% confidence intervals from the OLS models

Finally, the country-by-country comparison shown in Fig. 3 found that this effect is most significant in Finland, where online-gambling community participation clearly compensated for the life satisfaction of problem gamblers who had weak offline relationships.

## Discussion

The aim of this study was to analyze whether excessive gambling predicts lower life satisfaction and the extent to which online-gambling community participation and alternatively offline belonging modify that association. In particular, we assessed the extent to which online communities can serve as a source of life satisfaction for problem gamblers who had poor offline relationships. Our study was grounded in life satisfaction research (Diener et al., 1985; Headey et al., 1993) and social psychological theories (e.g., Baumeister & Leary, 1995), as well as research on online communities (Huang et al., 2021; Sirola et al., 2019).

The results confirmed the Hypothesis 1: Problem gambling was negatively associated with life satisfaction. However, the association was indirect, primarily through psychological distress and offline relationships. This result emphasizes the accumulation of problems and thus the ramifications of addiction on an individual’s entire life. This finding supports previous studies showing that problem gambling is associated with several other social problems (e.g., Dowling et al., 2014; Edgren et al., 2014; Wood & Griffiths, 2007). However, it seems that gambling on its own does not cause dissatisfaction with life, but dissatisfaction occurs through other factors, such as poor social relationships and psychological distress.

We hypothesized (Hypothesis 2) that online-gambling community participation may increase life satisfaction. Moreover, we assumed that life satisfaction of excessive gamblers might be improved through online communities (Hypothesis 3). The results confirmed both hypotheses. We found that daily participation in gambling communities increased life satisfaction and the effect was more prominent among probable pathological gamblers when compared to other participants. The result is in line with previous research that has shown active participation in online communities related to lifestyle and hobbies, as well as those providing social support, is linked to higher subjective well-being (Huang et al., 2019; Liu et al., 2020; Mahan et al., 2015; Oh et al., 2014).

Problem gambling is related to numerous other problems, of which weak offline relationships became central in the first phase of the analysis. We examined the ways offline relationships modify the relationship between participation in online communities and life satisfaction. The results confirmed Hypothesis 4: Online-gambling community participation improves the life satisfaction of problem gamblers, especially those with poor offline relationships. This result might indicate that online communities built around gambling offer problem gamblers a sense of social belonging and interaction they lack offline. This finding is in line with previous research showing an association between problem gambling and loneliness; it has been found that gambling-related online communities are particularly popular with players experiencing loneliness (Sirola et al., 2019).

In addition to the hypotheses, we also analyzed country differences. We found that the relationship between gambling and life dissatisfaction was significant only in Finland. We also found that the relationship between poor offline relationships and gambling was particularly strong in Finland. These results could reflect the current gambling situation among youths in Finland, where gambling prevalence is particularly high among 15 to 28-year-old adolescents and young adults. Around 18% of youths in this age group qualify as at-risk gamblers (Edgren et al., 2016). Our result suggests these Finnish youths might have specifically weak relationships offline. On the other hand, the result can also be an indication that online communities provide a platform for discussing and addressing problematic behavior that needs to be hidden from other close relations.

Internet-based gambling offers a potential environment for responsible gambling, including various player-focused tools such as expenditure tracking, self-set spend limits, time outs, and information on gambling problems (Gainsbury, 2015). This study has additionally shown that online gambling-community participation can increase the life satisfaction of problem gamblers. Although primarily used for facilitating gambling purposes, online communities also have the potential to serve as support networks to address individuals’ problems. In particular, it is vital to highlight the fact that the internet and various online communities make it possible to deal with problematic behavior effectively (Huang et al., 2019; Tanis, 2007). The anonymous nature of online platforms facilitates talking about one’s gambling-related problems and emotions and diminishes fear of being rejected or misunderstood. Thus, online communities can provide an opportunity for social interaction and support, especially for those with poor offline relationships and those who experience social stigma related to such issues as gambling problems. Although online contacts may not fully compensate for a lack of meaningful offline relationships, online networks can provide important social capital and a sense of belonging for lonely individuals, thus increasing well-being. Eventually, increased social capital may reduce the risk of problem gambling (Churchill & Farrell, 2020).

Findings of this study support the idea that various online communities may also be useful in reducing problem gambling at the population level. Although participation in gambling-related communities does not directly reduce gambling (Sirola et al., 2020), online communities generally can reduce experiences of loneliness and thus improve subjective well-being, which in turn can reduce the risks associated with problem gambling. It is also important to note that not all participants in gambling communities are problem gamblers. Thus, communities can also provide positive examples and peer influence for problem gamblers. There is also some evidence that critical feedback from online-gambling community members may help to reduce excessive gambling behavior (Parke & Griffiths, 2011). Thus, community and its peer influence may also have a protective role, as long as the community’s norms are in line with moderate forms of gambling.

## Limitations

Naturally, our study has its limitations. First, we could not test the causal inferences because the data were collected cross-sectionally. Therefore, we suggest that future studies utilize longitudinal data to investigate whether the associations exist over time. Second, the results of our study are potentially affected by social desirability bias because they rely on self-reported information on deviant behavior, such as problem gambling (Kuentzel et al., 2008). In this respect, experimental designs should be used to obtain estimates of the parameters in question that are more precise.

## Conclusion

The study examines the ways problem gambling as well as the use of gambling-related online communities are associated with young people’s life satisfaction in Finland, the United States, South Korea, and Spain. According to the results, problem gambling predicts lower life satisfaction, particularly in Finland. In contrast, participating in gambling-related online communities had a positive relationship with young people’s life satisfaction. We also found that the use of gambling-related online communities might increase problem gamblers’ life satisfaction, especially if they experience poor offline relationships. The results are generally consistent with previous literature on problem gamblers’ quality of life and the importance of online communities for life satisfaction. Problem gamblers often experience a variety of social problems, which can be alleviated by online communities formed around shared interests and potential support.

## Appendix

See Table

**Table 4:** Country interactions when predicting life satisfaction according to online gambling behavior and confounding variables

	Model 1		Model 2	
*Gambling problem*	*B*		*B*	
No risk (omitted)				
At risk	− 0.27*	(0.19)	− 0.27*	(0.15)
Probable pathological	− 0.95***	(0.22)	− 0.95***	(0.17)
*Interaction effects:*				
Probable pathological * Finland (omitted)				
Probable pathological * US	0.74*	(0.31)	0.54*	(0.23)
Probable pathological * South Korea	0.87*	(0.39)	0.66*	(0.30)
Probable pathological * Spain	0.88**	(0.28)	0.60**	(0.22)
*Country effects:* Finland (omitted)				
US	− 0.03	(0.08)	− 0.13	(0.08)
South Korea	− 0.92***	(0.07)	− 0.94***	(0.07)
Spain	− 0.05	(0.08)	0.30***	(0.08)
Constant	4.42***	(0.06)	4.42***	(0.06)
Control variables	NO		YES	
Observations	4,816		4,816	
R-squared	0.03		0.43	

Model 2 controls for the effects of age, gender, income, hazardous drinking, psychological distress, compulsive internet use and offline belonging

Standard errors in parentheses

^*^ p < 0.05

^**^ p < 0.01

^***^ p < 0.001

^4^.
